# Nerve Transfer for Triceps Reinnervation in Obstetrical Brachial Plexus Injury: Long-Term Functional Results

**DOI:** 10.1055/a-2837-4190

**Published:** 2026-04-09

**Authors:** Filippo M. Sénès, Francesca Susini, Maria Grazia Calevo, Chiara Arrigoni, Nunzio Catena

**Affiliations:** 1 Senior Medical Consultant of Hand Surgery Department, San Giuseppe Hospital, IRCCS Multimedica, Milan, Italy; 2 Hand Surgery Department, San Giuseppe Hospital, IRCCS Multimedica, Milan, Italy; 3 Epidemiology and Biostatistics Unit, Scientific Direction, IRCCS Institute Giannina Gaslini, Genoa, Italy; 4 Hand Surgery and Microsurgery Unit of IRCCS Istituto Giannina Gaslini, Genoa, Italy

**Keywords:** microsurgery, nerve transfers, obstetric brachial plexus injury, triceps reanimation, elbow flexion contracture

## Abstract

**Background:**

Restoration of elbow extension in obstetric brachial plexus injury (OBPI) with triceps paralysis is essential for optimizing upper limb function, although often considered a secondary objective.

**Objective:**

To assess long-term functional outcomes of triceps reinnervation via nerve transfers in pediatric OBPI patients with persistent triceps paralysis.

**Methods:**

This retrospective cohort included 16 patients who underwent triceps reanimation between 2007 and 2018. Inclusion criteria were the absence of active elbow extension and a minimum 4-year follow-up. Depending on the lesion level, nerve transfers involved motor branches of the ulnar nerve to the flexor carpi ulnaris (FCUm) and intercostal nerves. Descriptive notes of these procedures are provided in the text. Outcomes were evaluated using the Medical Research Council (MRC) scale for strength and Gilbert's Elbow Scale (GES) for function.

**Results:**

Mean age at surgery was 48.9 months (range: 8–96), with a minimum follow-up of 4 years. At final evaluation, triceps strength improved significantly (mean MRC: 3.75, median: 4) as did elbow function (mean: GES 3.75, median: 4) compared with preoperative values ( *p* < 0.01). Triceps recovery contributed to a mean 35-degree reduction in elbow flexion contracture, suggesting a preventive role against progressive deformity. No complications or donor site morbidity were observed.

**Conclusion:**

Delayed nerve transfers for triceps reinnervation—performed up to 8 years of age—yielded satisfactory long-term results without compromising recovery. These findings support including elbow extension restoration among surgical priorities in OBPI management, even for complete brachial plexus palsies when suitable donor nerves remain available after primary reconstructions.

## Introduction

In cases of obstetric brachial plexus injury (OBPI), reanimating the triceps muscle has traditionally been viewed as a secondary goal, usually considered only after hand function, elbow flexion, and shoulder mobility have been addressed. 1 2 However, active elbow extension plays a key role in reaching, enhancing grip, and maintaining overall limb stability.

Several approaches have been proposed to restore this function. 3 While muscle transfers are often used to recover shoulder motion and elbow flexion, they tend to be less reliable when it comes to restoring elbow extension. 4 5 6 In contrast, nerve transfers have shown more promise, with techniques involving the phrenic nerve, spinal accessory nerve, intercostal nerves, the medial pectoral nerve, ulnar nerve fascicles to the flexor carpi ulnaris muscle (FCUm), and even the contralateral C7 root. 7 8

Among these, the most consistently effective methods involve using intercostal nerves—particularly the third to fifth—or fascicles of the ulnar nerve that supply the FCUm. 9 10 11 12 13 In this study, we present long-term outcomes of triceps muscle reinnervation using nerve transfers in OBPI patients.

The primary objective of this project is to restore balance to elbow function by enhancing triceps muscle strength. An additional aim is to counteract the progression of elbow flexion contracture during growth. 14 15

## Methods

This is a retrospective study evaluating the long-term results of nerve transfer procedures for triceps reanimation in OBPI patients who underwent surgery between 2007 and 2018.

Inclusion criteria included patients with no triceps function from birth or those with persistent paralysis after failed radial nerve recovery, and a minimum of 4 years of follow-up after surgery. Nerve transfer performed for triceps muscle reanimation was directed toward the terminal branches of the radial nerve for the triceps muscle or toward the entire radial nerve.

Exclusion criteria were complete triceps denervation and concomitant weak elbow flexion strength less than M3 on the Medical Research Council (MRC) scale.

All patients underwent thorough preoperative evaluations, including clinical exams, imaging (ultrasound or MRI) to assess muscle quality, especially fatty degeneration, and electrophysiological studies to identify denervation. 16

Donor nerves were selected based on the type of plexus injury and the presence of active wrist flexion. Options included ulnar nerve fascicles to the FCUm and intercostal nerves, usually the third to fifth.

Surgery was performed under general anesthesia without muscle relaxants. Intraoperative electrical stimulation was used to guide fascicle selection—nonfunctioning fascicles were selected for transfer, while those with clear contractions were preserved.

## Surgical Techniques

Ulnar Nerve Fascicles Transfer to Radial Nerve (Oberlin-like Technique)

In this technique, fascicles of the ulnar nerve, specifically those innervating the FCUm muscle, are redirected to the nerve branches supplying the long head of the triceps muscle. For the first patient, the ulnar nerve was carefully dissected at the proximal third of the arm to harvest two appropriate fascicles. These fascicles were then routed through the axillary fold to reach the branches of the triceps muscle using a posterior surgical approach. In this instance, sural nerve grafts were required to bridge the gap to the target site.

In subsequent cases, except for one patient who also required nerve grafts, the procedure was refined to allow simultaneous exposure of both the ulnar and radial nerves within the axillary fold. This approach enabled a direct nerve suture to be performed without the need for grafting, streamlining the process and potentially reducing recovery time.

### Intercostal Nerves Transfer to Radial Nerve and Long Head of Triceps

For this procedure, three intercostal nerves are harvested using a direct approach at the level of the anterior axillary fold. The incision is made following a line from the anterior pillar of the armpit to the lower insertion of the pectoralis major muscle, typically spanning approximately 6 to 7 cm. Careful dissection is performed to expose the third, fourth, and fifth ribs while minimizing the length of the incision and avoiding unnecessary separation of the ribs.

The intercostal nerves are harvested via subperiosteal dissection through the muscle layers. Once an adequate length has been achieved, these nerves are transferred to the axilla for direct coaptation with the radial nerve. In practice, direct coaptation was successfully completed in three out of six patients, accounting for 50% of cases, so nerve grafts are likely required.

### Spinal Accessory Nerve to Radial Nerve Transfer

This approach utilizes a transverse incision across the antero-lateral triangle of the neck to expose the spinal accessory nerve using a classic method targeting the supraclavicular plexus. After identifying the anterior edge of the trapezius muscle, gentle retraction of the muscle enables the surgeon to visualize the nerve as it crosses the surgical field and descends toward the thorax.

Following the course of the nerve, careful distal dissection is undertaken to minimize denervation of the trapezius muscle. The radial nerve and the stump of the accessory spinal nerve are then joined via coaptation, which is accomplished through a subclavicular tunnel. Sural nerve grafts are used to bridge the distance to the axillary fold, ensuring the nerves are securely connected.

### Outcome Assessment

Outcomes were assessed using the MRC scale and Gilbert's Elbow Scale (GES). MRC scores were grouped as good (M4 or higher), fair (M3+ to M4 − ), or poor (below M3). GES outcomes were categorized as poor (grade I: 0–1 points), average (grade II: 2–3 points), or good (grade III: 4–5 points), with grade III considered functionally useful. This classification system is based on a cumulative scoring approach that quantifies the degree of muscle function in both flexion and extension, as well as the extent of extension defect.

For active flexion, the absence or hint of flexion is defined as a score of 0; incomplete flexion as a 2; and efficient flexion as a 3. For extension, when absent it is scored 0, weak as 1, and valid as 2. The extension defect is classified with a score of 0 if less than 30 degrees, as −1 if between 30 and 50 degrees, and as −2 above 50 degrees.

We analyzed age at surgery, duration of follow-up, age at final follow-up, and degree of elbow extension limitation, taking also into consideration elbow active flexion before surgery to ensure transparency and facilitate understanding of the surgical variables involved.

### Statistical Analyses Included Both Parametric and Nonparametric Tests

Continuous variables were expressed as mean ± standard deviation (SD) or as median and range. Categorical variables were reported as absolute and relative frequencies. Data distribution was assessed using the Kolmogorov–Smirnov test. Group comparisons were performed using the Wilcoxon test for continuous variables, and the *χ* ^2^ test or Fisher's exact test for categorical variables.

A *p* -value < 0.05 was considered statistically significant, and all *p* -values were based on two-tailed tests. Statistical analysis was performed using SPSS for Windows version 29 (SPSS Inc., Chicago, Illinois, United States).

## Results

From 2007 to 2018, 92 nerve transfer procedures were performed, 16 of which specifically involved triceps reinnervation. Patient demographics are summarized in Table 1 . The cohort showed equal gender distribution, with right-sided injuries in most cases. Eleven patients had C5–C6–C7 injuries. Three patients did not precisely match the classical presentation of C5–C6–C7 palsy because they exhibited persistent triceps muscle weakness, despite retaining active wrist and finger extension; therefore, we recognize that they should have been classified as C5–C6–(C7), with an explanatory note and C7 indicated in brackets to distinguish this subgroup from the broader C5–C6–C7 group. Considering the heterogeneity of our patient cohort, we have compiled a comprehensive table that details the preoperative and postoperative status of each case. This addition is intended to provide greater clarity regarding individual outcomes and to allow for more nuanced assessment of our results ( Table 2 ).

**Table 1:** Patient demographics and injury characteristics ( *n* = 16)

Characteristic	Category	*n* (%)
Gender	Male	8 (50%)
	Female	8 (50%)
Side of involvement	Right	15 (93.8%)
	Left	1 (6.3%)
Type of paralysis	C5–C6–(C7)	3 (18.8%)
	C5–C6–C7	11 (68.8%)
	C5–T1 (complete)	2 (12.5%)
Donor nerves used	Third–fifth intercostal nerves	6 (37.5%)
	FCU fascicles–ulnar nerve	9 (56.3%)
	Spinal accessory nerve (XI)	1 (6.3%)

Note: Summary of demographic data, side and type of paralysis, and donor nerves used in the cohort of 16 patients undergoing triceps reinnervation for obstetric brachial plexus injury.

**Table 2:** Summary of the demographic details, clinical features, and surgical procedures on a single patient

Case	Age (mo)	Sex	Side	Involved roots	Nerve transfer procedure	Active elbow flexion/GES (preoperative)	Triceps MRC (pre/final)	GES (pre/final)	Elbow extension limitation (degrees; final)
1	86	F	R	C5–C6–C7	U–R (CLTr)	2	0/3	1/3	40
2	67	M	L	C5–C6–C7	U–R (CLTr)	3	1/4	3/5	20
3	72	M	R	C5–C6–C7	IC–R (CLTr)	3	0/4	2/4	40
4	48	M	R	C5–C6–(C7)	U–R (CLTr)	3	0/4	3/5	20
5	36	F	R	C5–C6–C7	IC–R (graft) + SAN–R + SR	2	0/4	2/3	30
6	96	F	R	C5–C6–C7	IC–R (CLTr) + Hoffer	3	1/4	3/5	30
7	60	F	R	C5–C6–C7	U–RAD + TD–TL + SR	3	0/4	2/4	40
8	66	M	R	C5–C6–C7	SAN–SSN + U–RAD + SR	3	0/4	3/4	20
9	60	F	R	C5–C6–C7	SAN–AXC + U–RAD	3	0/3	3/3	30
10	36	M	R	C5–C6–(C7)	SAN–SSN + U–RAD + SR	2	1/4	3/3	50
11	48	M	R	C5–T1	SAN–RAD + IC–RAD + SR	3	0/3	2/4	40
12	36	F	R	C5–C6–(C7)	SAN–SSN + U–RAD + SR	3	0/3	2/3	40
13	12	F	R	C5–C6–C7	SAN–SSN + U–RAD + SR	2	1/5	3/3	20
14	16	M	R	C5–C6–C7	IC–RAD	3	0/2	1/2	60
15	8	F	R	C5–T1	SAN–SSN + IC–RAD	2	0/5	3/5	20
16	36	M	R	C5–C6–C7	IC–RAD	3	0/4	2/4	40

Abbreviations: GES, Gilbert's elbow scale; Hoffer, latissimus dorsi muscle transfer to rotator cuff; IC, intercostal nerves; MRC, Medical Research Council scale; RAD, radial nerve; RNBLHT, Radial nerve branches to long head of triceps m.; SAN, spinal accessory nerve; SR, Subscapularis muscle release; SSN, suprascapular nerve; TD–LT, thoracodorsal to long thoracic nerve transfer; U, ulnar nerve.

Surgical techniques included:

- Six patients receiving intercostal nerve transfers (third–fifth).

- Nine receiving ulnar nerve fascicle transfers to the FCUm.

- One patient receiving a spinal accessory nerve transfer to the triceps, combined with intercostals, to restore wrist extension.

- Seven patients undergoing combined procedures to restore both triceps and shoulder function to reduce shoulder stiffness and improve external rotation.

The mean age at surgery was 48.94 months (median: 48; range: 8–96 months). Follow-up ranged from 4 to 13 years (mean: 9; median: 9.57). At final follow-up, the mean patient age was 13 years (median: 12.56; range: 5.5–18.1). Most patients were over 11 years old; three patients (18%) were under 10. All patients had a minimum follow-up of 4 years.

Limitation of elbow extension is commonly referred to as an elbow flexion contracture, although the actual joint restriction pertains to extension rather than flexion.

At final follow-up, elbow extension was limited in all patients, with a median contracture angle of 35 degrees, which was considered stable due to the long follow-up. ( Table 3 ).

**Table 3:** Surgical timing, follow-up duration, and elbow extension limitation

Parameter	Mean	Median	SD	Range
Age at surgery (mo)	48.94	48.00	25.53	8–96
Follow-up duration (ΔT, ye)	8.76	9.57	3.36	4.07–13.10
Age at last follow-up (y)	12.84	12.56	3.50	5.46–18.10
Elbow extension limitation (degree)	33.75	35.00	12.04	20–60

Note: Descriptive statistics for age at surgery, follow-up duration (ΔT), age at last follow-up, and degree of elbow extension limitation in the study cohort.

Before surgery, only 25% of patients had minimal triceps activity (MRC grade: 1), while the rest had none (grade: 0). At final follow-up, the mean MRC score was 3.75 (range: 2–5), with a median of 4. GES scores improved from a preoperative mean of 2.38 (range: 1–3; median: 2.50) to a postoperative mean of 3.75 (range: 2–5; median: 4; Table 4 ).

**Table 4:** Functional outcomes: MRC and GES scores

Scale	Mean	Median	SD	Range
MRC score: preoperative	0.25	0.00	0.45	0–1
MRC score: final follow-up	3.75	4.00	0.77	2–5
GES score: preoperative	2.38	2.50	0.72	1–3
GES score: final follow-up	3.75	4.00	0.93	2–5

Abbreviations: GES, Gilbert Elbow scale; MRC, Medical Research Council scale; SD, standard deviation.

Note: Preoperative and postoperative Medical Research Council (MRC) and Gilbert's Elbow Scale (GES) scores, showing improvements in triceps strength and elbow function following nerve transfer procedures.

These improvements were statistically significant in both MRC and GES scores postoperatively ( *p* < 0.001).

There was no significant difference in outcomes between patients who had nerve transfers as part of their primary OBPI surgery and those who had no prior plexus repair. Importantly, harvesting intercostal nerves did not lead to chest wall deformities, likely due to limited and careful surgical exposure.

## Discussion

In OBPI, an imbalance between weak elbow extensors and relatively stronger flexors can lead to joint deformities and progressive contractures, resulting in the elbow becoming fixed in a flexed position due to periarticular tissue stiffening. This restricted extension is known as an elbow flexion contracture.

**Fig. 1:**
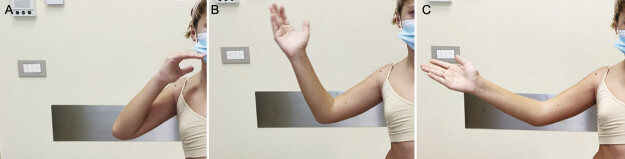
Active range of motion of elbow extension in a patient with triceps muscle palsy caused by a C5–C6–C7 injury. The patient underwent muscle reinnervation using ulnar nerve fascicles. The sequence shows complete flexion ( **A** ), maintenance of a semi-flexed position ( **B** ), and complete extension of the elbow ( **C** ).

**Fig. 2:**
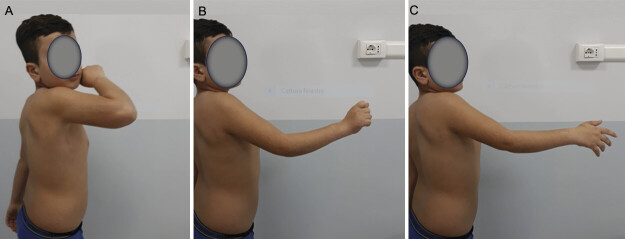
( **A** ) Shows active elbow extension after intercostal nerve transfer in a patient who had persistent triceps muscle palsy despite undergoing previous primary brachial plexus reconstruction. ( **B** ) Demonstrates simultaneous elbow and wrist extension. ( **C** ) Illustrates elbow extension with finger extension.

Our results show that reinnervating the triceps via nerve transfer leads to functional gains in upper limb use. Our selection of surgical strategy was primarily determined by the level of palsy and the functional status of the ulnar nerve. In cases of C5–C6–C7 palsy where ulnar nerve activity was preserved, we opted for a transfer of ulnar nerve fascicles to the triceps muscle ( Fig. 1 ). Conversely, for patients in whom the ulnar nerve was impaired, we pursued intercostal nerve transfers as an alternative approach ( Fig. 2 ).

In more severe cases, the initial surgical goal should always be restoration of essential functions—like hand grip, elbow flexion, and shoulder stability. Still, when reliable donor nerves are available, triceps reanimation can be a valuable addition.

Our findings reinforce the existing evidence supporting intercostal nerve transfers 9 10 and show that meaningful motor recovery is achievable.

Functional outcomes in this cohort demonstrated significant improvements in both triceps muscle strength and elbow extension. Median GES and MRC scores reached functionally useful levels postoperatively, with GES at 4 and MRC at M4, indicating recovery of strength against resistance and meaningful clinical benefit.

Although early nerve Surgery—ideally performed between 3 and 9 months of age—is preferred, our data show that delayed nerve transfers can still yield good outcomes, especially in patients without osteoarticular deformities. In OBPI, complete muscle denervation is rare; residual electrical activity, often due to neuromas in continuity, may preserve muscle viability even without visible movement. Electromyographic evidence of late fibrillation potentials supports ongoing muscle viability, likely sustained by residual impulses through neuromas in continuity. 15

Patients in our cohort who underwent delayed surgery still achieved satisfactory recovery. So, while age is a factor, it should not necessarily be a strict exclusion criterion. That said, the extent to which triceps recovery prevents elbow contracture is still unclear, as many factors contribute to contracture formation. Notably, we observed no joint deformities or soft tissue issues that would limit recovery.

This study has several inherent limitations, including its retrospective design, small sample size, and lack of a control group. Nonetheless, the long-term follow-up and consistent functional assessments strengthen the clinical relevance of the findings. The heterogeneity of patients and surgical techniques reflects real-world OBPI variability, supporting the adaptability of nerve transfer approaches. While the impact of triceps reinnervation on contracture prevention remains to be confirmed, the absence of deformity progression at follow-up is promising. Further prospective studies, including patient-reported outcomes, are needed to validate these results and better define their functional impact.

## Conclusion

The findings of this study support the role of selective nerve transfers as a valuable option for triceps muscle reanimation in children with OBPI. Despite being traditionally considered a secondary goal, restoring active elbow extension contributes meaningfully to upper limb function, particularly in terms of reach, limb positioning, and grip optimization.

Our results demonstrate consistent functional improvement, with statistically and clinically significant gains in both MRC and GES scores, sustained over long-term follow-up. Importantly, triceps neurotization proved feasible across a range of patient ages and injury patterns, reinforcing the versatility of this approach.

The absence of thoracic deformities following intercostal nerve harvesting and the preservation of donor site function further support the safety and applicability of the technique.

While the precise role of triceps reinnervation in preventing elbow contractures remains to be fully clarified, the stable joint outcomes observed in our cohort are encouraging. Future prospective studies with larger cohorts and standardized, patient-centered outcome measures are warranted to better define the long-term functional and developmental impact of triceps reanimation.

Nevertheless, this work provides meaningful evidence to guide surgical decision-making and underscores the importance of considering elbow extension as a functional priority in the comprehensive treatment of OBPI.

## References

[1] Bertelli J Soldado F Ghizoni M F Rodríguez-Baeza A Transfer of a terminal motor branch nerve to the flexor carpi ulnaris for triceps muscle reinnervation: anatomical study and clinical cases J Hand Surg Am 2015 40 11 2229 223500 26433244 10.1016/j.jhsa.2015.08.014

[2] Bertelli J A Lower trapezius muscle transfer for reconstruction of elbow extension in brachial plexus injuries J Hand Surg Eur Vol 2009 34 04 459 464 19587075 10.1177/1753193408101466

[3] Bonds C W James M A Posterior deltoid-to-triceps tendon transfer to restore active elbow extension in patients with tetraplegia Tech Hand Up Extrem Surg 2009 13 02 94 97 19516135 10.1097/BTH.0b013e318196c92d

[4] El-Gammal T A El-Sayed A Kotb M M Saleh W R Ragheb Y F el-Refai O Delayed selective neurotization for restoration of elbow and hand functions in late presenting obstetrical brachial plexus palsy J Reconstr Microsurg 2014 30 04 271 274 24696398 10.1055/s-0033-1357280

[5] Flores L P Transfer of a motor fascicle from the ulnar nerve to the branch of the radial nerve destined to the long head of the triceps for restoration of elbow extension in brachial plexus surgery: technical case report Neurosurgery 2012 70 02 E516 E520, discussion E520 21795861 10.1227/NEU.0b013e31822ac120

[6] Gilbert A Tassin J L Réparation chirurgicale du plexus brachial dans la paralysie obstétricale Chirurgie 1984 110 01 70 75 6734350

[7] Goubier J N Maillot C Asmar G Teboul F Partial ulnar nerve transfer to the branch of the long head of the triceps to recover elbow extension in C5, C6 and C7 brachial plexus palsy Injury 2019 50 05 S68 S70 31690498 10.1016/j.injury.2019.10.052

[8] Goubier J N Teboul F Khalifa H Reanimation of elbow extension with intercostal nerves transfers in total brachial plexus palsies Microsurgery 2011 31 01 7 11 21207492 10.1002/micr.20822

[9] Goubier J N Teboul F Transfer of the intercostal nerves to the nerve of the long head of the triceps to recover elbow extension in brachial plexus palsy Tech Hand Up Extrem Surg 2007 11 02 139 141 17549019 10.1097/bth.0b013e31803105e1

[10] Hale H B Bae D S Waters P M Current concepts in the management of brachial plexus birth palsy J Hand Surg Am 2010 35 02 322 331 20141905 10.1016/j.jhsa.2009.11.026

[11] Nehete S Bertelli J A Lower trapezius muscle transfer for elbow extension reconstruction after failed nerve transfer for tetraplegia J Hand Surg Am 2020 45 06 5580 5.58E6 10.1016/j.jhsa.2019.07.016 31585742

[12] Oberlin C Béal D Leechavengvongs S Salon A Dauge M C Sarcy J J Nerve transfer to biceps muscle using a part of ulnar nerve for C5-C6 avulsion of the brachial plexus: anatomical study and report of four cases J Hand Surg Am 1994 19 02 232 237 8201186 10.1016/0363-5023(94)90011-6

[13] Sénès F Catena N Sénès J Nerve transfer in delayed obstetrical palsy repair J Brachial Plex Peripher Nerve Inj 2015 10 01 e2 e14 27917233 10.1055/s-0035-1549367 PMC5023088

[14] Senes F M Catena N Dapelo E Senes J Nerve transfer for elbow extension in obstetrical brachial plexus palsy Ann Acad Med Singap 2016 45 05 221 224 27383724

[15] Terzis J K Kokkalis Z T Restoration of elbow extension after primary reconstruction in obstetric brachial plexus palsy J Pediatr Orthop 2010 30 02 161 168 20179564 10.1097/BPO.0b013e3181cf2e82

[16] Serhal A Lee S K Michalek J Serhal M Omar I M Role of high-resolution ultrasound and magnetic resonance neurography in the evaluation of peripheral nerves in the upper extremity J Ultrason 2023 23 95 e313 e327 38020515 10.15557/jou.2023.0037 PMC10668945

